# *N*,*N*,*N*′,*N*′,*N*′′-Penta­methyl-*N*′′-[3-(1,3,3-trimethyl­ureido)prop­yl]guanidinium tetra­phenyl­borate

**DOI:** 10.1107/S1600536812027067

**Published:** 2012-06-27

**Authors:** Ioannis Tiritiris, Willi Kantlehner

**Affiliations:** a Institut für Organische Chemie, Universität Stuttgart, Pfaffenwaldring 55, 70569 Stuttgart, Germany; b Fakultät Chemie/Organische Chemie, Hochschule Aalen, Beethovenstrasse 1, D-73430 Aalen, Germany

## Abstract

In the crystal structure of the title molecular salt, C_13_H_30_N_5_O^+^·C_24_H_20_B^−^, discrete guanidinium cations and tetra­phenyl­borate anions are present. The C—N bond lengths in the CN_3_ unit are 1.3427 (12), 1.3445 (12) and 1.3453 (13) Å, indicating double-bond character. The central C atom is surrounded in a nearly ideal trigonal-planar geometry by three N atoms and the positive charge is delocalized on the CN_3_ plane. The bonds between the N atoms and the terminal C-methyl groups all have values close to a typical single bond [1.4595 (15)–1.4688 (12) Å]. In the crystal, cations are connected by C—H⋯O contacts generating a chain along the *c* axis.

## Related literature

For the synthesis of 1-methyl- 2-dimethyl­amino-1,4,5,6-tetra­hydro­pyrimidine and *N*′′-[3-(1,3,3-trimethyl­ureido)prop­yl]-*N*,*N*,*N*′,*N*′,*N*′′-tetra­methyl­guanidine and derived guanidinium salts, see: Tiritiris & Kantlehner (2012 ▶). For the crystal structure of *N*,*N*,*N*′,*N*′- tetra­methyl­chloro­formamidinium-chloride, see: Tiritiris & Kantlehner (2008 ▶) and of *N*,*N*,*N*′,*N*′-tetra­methyl­urea, see: Frampton & Parkes (1996 ▶).
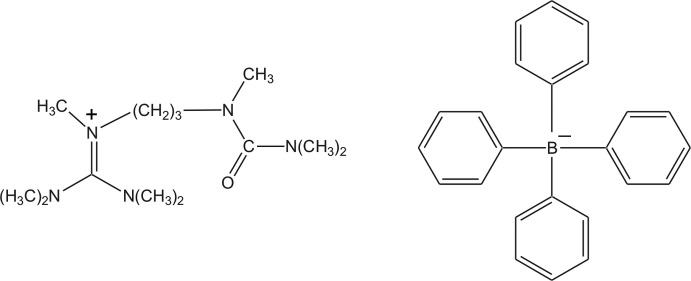


## Experimental

### 

#### Crystal data

C_13_H_30_N_5_O^+^·C_24_H_20_B^−^
*M* *_r_* = 591.63
Monoclinic, 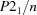

*a* = 16.6807 (7) Å
*b* = 9.7766 (4) Å
*c* = 21.6911 (9) Å
β = 110.743 (3)°
*V* = 3308.1 (2) Å^3^
*Z* = 4
Mo *K*α radiation
μ = 0.07 mm^−1^
*T* = 100 K
0.26 × 0.20 × 0.17 mm

#### Data collection

Bruker Kappa APEXII DUO diffractometer
69308 measured reflections
10094 independent reflections
7953 reflections with *I* > 2σ(*I*)
*R*_int_ = 0.037

#### Refinement

*R*[*F* ^2^ > 2σ(*F* ^2^)] = 0.041
*wR*(*F* ^2^) = 0.107
*S* = 1.01
10094 reflections
405 parameters
H-atom parameters constrained
Δρ_max_ = 0.39 e Å^−3^
Δρ_min_ = −0.22 e Å^−3^

### 

Data collection: *APEX2* (Bruker, 2008 ▶); cell refinement: *SAINT* (Bruker, 2008 ▶); data reduction: *SAINT*; program(s) used to solve structure: *SHELXS97* (Sheldrick, 2008 ▶); program(s) used to refine structure: *SHELXL97* (Sheldrick, 2008 ▶); molecular graphics: *DIAMOND* (Brandenburg & Putz, 2005 ▶); software used to prepare material for publication: *SHELXL97*.

## Supplementary Material

Crystal structure: contains datablock(s) I, global. DOI: 10.1107/S1600536812027067/kp2424sup1.cif

Structure factors: contains datablock(s) I. DOI: 10.1107/S1600536812027067/kp2424Isup2.hkl

Additional supplementary materials:  crystallographic information; 3D view; checkCIF report

## Figures and Tables

**Table 1:** Hydrogen-bond geometry (Å, °)

*D*—H⋯*A*	*D*—H	H⋯*A*	*D*⋯*A*	*D*—H⋯*A*
C13—H13*C*⋯O1^i^	0.98	2.67	3.395 (2)	131

Symmetry code: (i) 
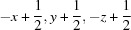
.

**Fig. 1.**
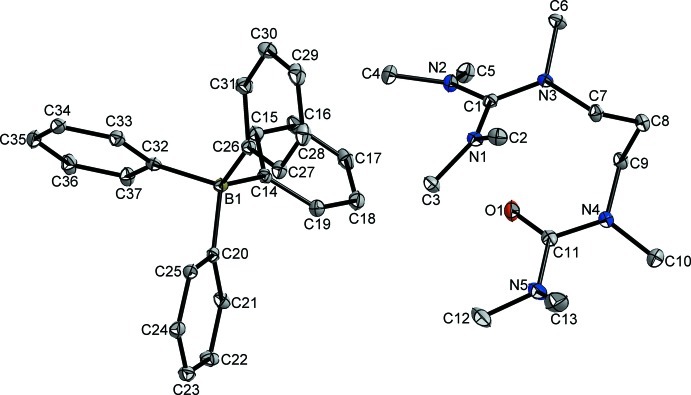
The structure of the title compound with atom labels and 50% probability displacement ellipsoids. All H atoms were omitted for clarity.

**Fig. 2.**
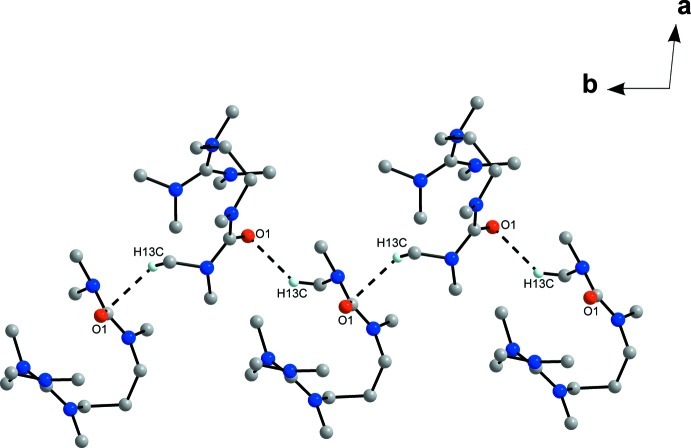
C–H···O contacts between the guanidinium ions, ab-view. The contacts are indicated by dashed lines.

## supplementary crystallographic information

### Comment

In the synthesis of the cyclic guanidine compound 1-methyl- 2-dimethylamino-1,4,5,6-tetrahydropyrimidine (Tiritiris & Kantlehner, 2012) from *N*,*N*,*N*',*N*'-tetramethylchloroformamidinium- chloride (Tiritiris & Kantlehner, 2008) and *N*-methyl-propane-1,3-diamine, the guanidine-urea derivative *N*''-[3-(1,3,3-Trimethylureido)propyl]- *N*,*N*,*N*',*N*',*N*''-tetramethylguanidine was obtained as a byproduct. By alkylation of the free nitrogen of the guanidine moiety, a few guanidinium salts have been obtained and structurally characterised (Tiritiris & Kantlehner, 2012). One of them, is the here presented title compound. In the crystal structure of the salt, isolated cations and anions are present. No specific interactions between the guanidinium ions and the tetraphenylborate ions have been observed. Prominent bond parameters in the guanidinium ion are: C1–N1 = 1.345 (1) Å, C1–N2 = 1.343 (1) Å and C1–N3 = 1.345 (1) Å. The N–C1–N angles are: 119.65 (9)° (N1–C1–N2), 119.57 (9)° (N2–C1–N3) and 120.78 (9)° (N1–C1–N3), which indicate a nearly ideal trigonal-planar surrounding of the carbon centre by the nitrogen atoms. The positive charge is completely delocalised in the CN_3_ plane (Fig. 1). Bond lengths in the ureido group are: C11–O1 = 1.229 (1) Å, C11–N4 = 1.370 (1) Å and C11–N5 = 1.389 (1) Å. These values agree very well with the data from the crystal structure analysis of solid *N*,*N*,*N*',*N*'-tetramethylurea (Frampton & Parkes, 1996). Finally, C–H···O contacts between methyl hydrogen atoms and carbonyl oxygen atoms of neighbouring guanidinium ions have been observed [*d*(H···O) = 2.67 Å] (Tab. 1), generating a chain (Fig. 2). The anions are packed inbetween these chains by van der Waals interactions.

### Experimental

The title compound was obtained by reaction of *N*''-[3-(1,3,3-Trimethylureido)propyl]- *N*,*N*,*N*',*N*',*N*''-tetramethylguanidine with dimethyl sulfate in acetonitrile at room temperature. After evaporation of the solvent the crude *N*''-[3-(1,3,3-Trimethylureido)propyl]-*N*,*N*,*N*', *N*',*N*''-pentamethylguanidinium-methylsulfate (I) was washed with diethylether and dried *in vacuo*. 1.00 g (2.6 mmol) of (I) was dissolved in 20 mL acetonitrile and 0.89 g (2.6 mmol) of sodium tetraphenylborate in 10 mL acetonitrile was added. After stirring for one h at room temperature, the precipitated sodium methylsulfate was filtered off. The title compound crystallised from a saturated acetonitrile solution after several days at 273 K, forming colourless single crystals. Yield: 1.18 g (76.9%).

### Refinement

The hydrogen atoms of the methyl groups were allowed to rotate with a fixed angle around the C–N bond to best fit the experimental electron density, with *U*(H) set to 1.5 *U*_eq_(C) and d(C—H) = 0.98 Å. The remaining H atoms were placed in calculated positions with d(C—H) = 0.99 Å (H atoms in CH_2_ groups) and (C—H) = 0.95 Å (H atoms in aromatic rings). They were included in the refinement in the riding model approximation, with *U*(H) set to 1.2 *U*_eq_(C).

### Crystal data

**Table:**

C_13_H_30_N_5_O^+^·C_24_H_20_B^−^	*F*(000) = 1280
*M**_r_* = 591.63	*D*_x_ = 1.188 Mg m^−^^3^
Monoclinic, *P*2_1_/*n*	Melting point: 426 K
Hall symbol: -P 2yn	Mo *K*α radiation, λ = 0.71073 Å
*a* = 16.6807 (7) Å	Cell parameters from 10094 reflections
*b* = 9.7766 (4) Å	θ = 1.3–30.5°
*c* = 21.6911 (9) Å	µ = 0.07 mm^−^^1^
β = 110.743 (3)°	*T* = 100 K
*V* = 3308.1 (2) Å^3^	Polyhedral, colourless
*Z* = 4	0.26 × 0.20 × 0.17 mm

### Data collection

**Table:**

Bruker Kappa APEXII DUO diffractometer	7953 reflections with *I* > 2σ(*I*)
Radiation source: sealed tube	*R*_int_ = 0.037
Graphite monochromator	θ_max_ = 30.5°, θ_min_ = 1.3°
φ scans, and ω scans	*h* = −23→22
69308 measured reflections	*k* = −13→13
10094 independent reflections	*l* = −22→30

### Refinement

**Table:**

Refinement on *F*^2^	Primary atom site location: structure-invariant direct methods
Least-squares matrix: full	Secondary atom site location: difference Fourier map
*R*[*F*^2^ > 2σ(*F*^2^)] = 0.041	Hydrogen site location: difference Fourier map
*wR*(*F*^2^) = 0.107	H-atom parameters constrained
*S* = 1.01	*w* = 1/[σ^2^(*F*_o_^2^) + (0.0496*P*)^2^ + 1.1611*P*] where *P* = (*F*_o_^2^ + 2*F*_c_^2^)/3
10094 reflections	(Δ/σ)_max_ < 0.001
405 parameters	Δρ_max_ = 0.39 e Å^−^^3^
0 restraints	Δρ_min_ = −0.22 e Å^−^^3^

### Special details

**Table:**

Geometry. All e.s.d.'s (except the e.s.d. in the dihedral angle between two l.s. planes) are estimated using the full covariance matrix. The cell e.s.d.'s are taken into account individually in the estimation of e.s.d.'s in distances, angles and torsion angles; correlations between e.s.d.'s in cell parameters are only used when they are defined by crystal symmetry. An approximate (isotropic) treatment of cell e.s.d.'s is used for estimating e.s.d.'s involving l.s. planes.
Refinement. Refinement of *F*^2^ against ALL reflections. The weighted *R*-factor *wR* and goodness of fit *S* are based on *F*^2^, conventional *R*-factors *R* are based on *F*, with *F* set to zero for negative *F*^2^. The threshold expression of *F*^2^ > σ(*F*^2^) is used only for calculating *R*-factors(gt) *etc*. and is not relevant to the choice of reflections for refinement. *R*-factors based on *F*^2^ are statistically about twice as large as those based on *F*, and *R*- factors based on ALL data will be even larger.

### Fractional atomic coordinates and isotropic or equivalent isotropic displacement parameters (Å^2^)

**Table:**

	*x*	*y*	*z*	*U*_iso_*/*U*_eq_	
C1	0.00142 (6)	0.43833 (10)	0.28523 (5)	0.01237 (18)	
N1	0.04589 (5)	0.54738 (9)	0.27727 (4)	0.01311 (16)	
N2	0.01497 (5)	0.39040 (9)	0.34611 (4)	0.01480 (16)	
N3	−0.05660 (5)	0.37668 (9)	0.23291 (4)	0.01248 (16)	
C2	0.00943 (6)	0.65341 (11)	0.22761 (5)	0.01589 (19)	
H2A	−0.0523	0.6378	0.2060	0.024*	
H2B	0.0371	0.6501	0.1946	0.024*	
H2C	0.0190	0.7434	0.2489	0.024*	
C3	0.13425 (6)	0.57032 (12)	0.32099 (5)	0.0181 (2)	
H3A	0.1354	0.6410	0.3533	0.027*	
H3B	0.1686	0.6003	0.2950	0.027*	
H3C	0.1580	0.4851	0.3440	0.027*	
C4	0.03473 (7)	0.48174 (12)	0.40317 (5)	0.0198 (2)	
H4A	0.0946	0.4689	0.4319	0.030*	
H4B	−0.0030	0.4605	0.4277	0.030*	
H4C	0.0259	0.5769	0.3880	0.030*	
C5	0.01339 (7)	0.24405 (11)	0.35967 (5)	0.0184 (2)	
H5A	−0.0403	0.2213	0.3662	0.028*	
H5B	0.0620	0.2211	0.3996	0.028*	
H5C	0.0174	0.1917	0.3223	0.028*	
C6	−0.13342 (6)	0.31226 (11)	0.23767 (5)	0.0168 (2)	
H6A	−0.1259	0.2128	0.2403	0.025*	
H6B	−0.1832	0.3356	0.1986	0.025*	
H6C	−0.1426	0.3453	0.2773	0.025*	
C7	−0.05146 (6)	0.38013 (10)	0.16672 (5)	0.01345 (18)	
H7A	0.0013	0.4294	0.1688	0.016*	
H7B	−0.1011	0.4318	0.1369	0.016*	
C8	−0.05061 (6)	0.23790 (11)	0.13824 (5)	0.01458 (18)	
H8A	−0.0525	0.2467	0.0923	0.017*	
H8B	−0.1026	0.1877	0.1373	0.017*	
C9	0.02876 (6)	0.15555 (10)	0.17809 (5)	0.01427 (18)	
H9A	0.0315	0.1485	0.2243	0.017*	
H9B	0.0239	0.0618	0.1599	0.017*	
N4	0.10752 (5)	0.21848 (9)	0.17680 (4)	0.01459 (17)	
C10	0.12466 (7)	0.20148 (12)	0.11575 (5)	0.0187 (2)	
H10A	0.0953	0.2736	0.0846	0.028*	
H10B	0.1037	0.1119	0.0965	0.028*	
H10C	0.1865	0.2075	0.1252	0.028*	
C11	0.16660 (6)	0.26634 (10)	0.23433 (5)	0.01496 (19)	
O1	0.16215 (5)	0.24141 (9)	0.28856 (4)	0.02149 (17)	
N5	0.23374 (6)	0.34149 (10)	0.22769 (5)	0.0218 (2)	
C12	0.30581 (7)	0.36738 (14)	0.28860 (7)	0.0307 (3)	
H12A	0.2910	0.4416	0.3130	0.046*	
H12B	0.3562	0.3937	0.2780	0.046*	
H12C	0.3186	0.2843	0.3156	0.046*	
C13	0.21641 (8)	0.45323 (13)	0.17992 (7)	0.0283 (3)	
H13A	0.1569	0.4474	0.1494	0.042*	
H13B	0.2552	0.4462	0.1551	0.042*	
H13C	0.2255	0.5410	0.2032	0.042*	
B1	0.27778 (7)	0.66069 (11)	0.56100 (5)	0.01149 (19)	
C14	0.25827 (6)	0.49830 (10)	0.54196 (4)	0.01180 (17)	
C15	0.22288 (6)	0.40787 (11)	0.57578 (5)	0.01462 (19)	
H15A	0.2108	0.4410	0.6128	0.018*	
C16	0.20468 (7)	0.27111 (11)	0.55717 (5)	0.0179 (2)	
H16A	0.1813	0.2133	0.5817	0.021*	
C17	0.22064 (7)	0.21925 (11)	0.50295 (5)	0.0192 (2)	
H17A	0.2096	0.1259	0.4908	0.023*	
C18	0.25315 (7)	0.30673 (12)	0.46684 (5)	0.0185 (2)	
H18A	0.2631	0.2737	0.4290	0.022*	
C19	0.27107 (6)	0.44254 (11)	0.48620 (5)	0.01493 (19)	
H19A	0.2929	0.5003	0.4606	0.018*	
C20	0.36740 (6)	0.71189 (10)	0.55261 (4)	0.01193 (17)	
C21	0.43511 (6)	0.62355 (11)	0.55543 (5)	0.01413 (18)	
H21A	0.4270	0.5278	0.5578	0.017*	
C22	0.51385 (6)	0.67076 (11)	0.55480 (5)	0.0166 (2)	
H22A	0.5577	0.6073	0.5565	0.020*	
C23	0.52831 (6)	0.81001 (11)	0.55176 (5)	0.0165 (2)	
H23A	0.5818	0.8427	0.5515	0.020*	
C24	0.46291 (6)	0.90079 (11)	0.54911 (5)	0.01526 (19)	
H24A	0.4717	0.9964	0.5471	0.018*	
C25	0.38455 (6)	0.85204 (11)	0.54939 (5)	0.01370 (18)	
H25A	0.3409	0.9162	0.5473	0.016*	
C26	0.19311 (6)	0.74476 (10)	0.51335 (5)	0.01287 (18)	
C27	0.18843 (6)	0.82027 (11)	0.45734 (5)	0.01507 (19)	
H27A	0.2381	0.8266	0.4457	0.018*	
C28	0.11366 (7)	0.88671 (11)	0.41778 (5)	0.0189 (2)	
H28A	0.1133	0.9368	0.3802	0.023*	
C29	0.03995 (7)	0.87970 (12)	0.43334 (5)	0.0215 (2)	
H29A	−0.0107	0.9260	0.4071	0.026*	
C30	0.04144 (7)	0.80373 (13)	0.48794 (5)	0.0220 (2)	
H30A	−0.0087	0.7967	0.4989	0.026*	
C31	0.11627 (6)	0.73815 (12)	0.52643 (5)	0.0180 (2)	
H31A	0.1156	0.6864	0.5633	0.022*	
C32	0.29464 (6)	0.69109 (10)	0.63942 (5)	0.01246 (18)	
C33	0.26863 (6)	0.81209 (11)	0.66204 (5)	0.01528 (19)	
H33A	0.2345	0.8758	0.6306	0.018*	
C34	0.29113 (7)	0.84212 (12)	0.72909 (5)	0.0186 (2)	
H34A	0.2725	0.9252	0.7423	0.022*	
C35	0.34055 (7)	0.75121 (12)	0.77639 (5)	0.0200 (2)	
H35A	0.3561	0.7715	0.8220	0.024*	
C36	0.36696 (7)	0.62986 (12)	0.75598 (5)	0.0190 (2)	
H36A	0.4002	0.5659	0.7877	0.023*	
C37	0.34463 (6)	0.60215 (11)	0.68907 (5)	0.01543 (19)	
H37A	0.3641	0.5192	0.6764	0.019*	

### Atomic displacement parameters (Å^2^)

**Table:**

	*U*^11^	*U*^22^	*U*^33^	*U*^12^	*U*^13^	*U*^23^
C1	0.0118 (4)	0.0119 (4)	0.0141 (4)	0.0013 (3)	0.0054 (3)	−0.0010 (3)
N1	0.0113 (3)	0.0124 (4)	0.0149 (4)	−0.0008 (3)	0.0037 (3)	−0.0006 (3)
N2	0.0171 (4)	0.0150 (4)	0.0122 (4)	−0.0007 (3)	0.0052 (3)	−0.0008 (3)
N3	0.0118 (4)	0.0127 (4)	0.0131 (4)	−0.0022 (3)	0.0046 (3)	−0.0007 (3)
C2	0.0164 (4)	0.0112 (5)	0.0205 (5)	0.0002 (4)	0.0072 (4)	0.0017 (4)
C3	0.0123 (4)	0.0204 (5)	0.0196 (5)	−0.0039 (4)	0.0035 (4)	−0.0042 (4)
C4	0.0218 (5)	0.0244 (6)	0.0143 (5)	−0.0031 (4)	0.0077 (4)	−0.0054 (4)
C5	0.0231 (5)	0.0165 (5)	0.0159 (5)	0.0006 (4)	0.0073 (4)	0.0032 (4)
C6	0.0131 (4)	0.0179 (5)	0.0200 (5)	−0.0042 (4)	0.0065 (4)	−0.0012 (4)
C7	0.0150 (4)	0.0131 (5)	0.0114 (4)	−0.0004 (3)	0.0036 (3)	0.0008 (3)
C8	0.0137 (4)	0.0154 (5)	0.0129 (4)	−0.0021 (4)	0.0026 (3)	−0.0024 (4)
C9	0.0142 (4)	0.0131 (5)	0.0148 (4)	−0.0016 (3)	0.0042 (4)	−0.0007 (4)
N4	0.0130 (4)	0.0173 (4)	0.0128 (4)	−0.0021 (3)	0.0038 (3)	−0.0025 (3)
C10	0.0202 (5)	0.0219 (5)	0.0149 (5)	−0.0011 (4)	0.0073 (4)	−0.0019 (4)
C11	0.0120 (4)	0.0125 (5)	0.0182 (5)	0.0033 (3)	0.0027 (4)	−0.0038 (4)
O1	0.0207 (4)	0.0264 (4)	0.0143 (4)	0.0011 (3)	0.0025 (3)	−0.0052 (3)
N5	0.0139 (4)	0.0202 (5)	0.0291 (5)	−0.0035 (3)	0.0047 (4)	−0.0070 (4)
C12	0.0138 (5)	0.0307 (7)	0.0418 (7)	−0.0025 (5)	0.0029 (5)	−0.0197 (6)
C13	0.0324 (6)	0.0196 (6)	0.0395 (7)	−0.0088 (5)	0.0207 (6)	−0.0057 (5)
B1	0.0122 (4)	0.0115 (5)	0.0111 (4)	−0.0009 (4)	0.0044 (4)	−0.0003 (4)
C14	0.0101 (4)	0.0133 (4)	0.0104 (4)	0.0006 (3)	0.0016 (3)	0.0002 (3)
C15	0.0142 (4)	0.0159 (5)	0.0130 (4)	−0.0026 (4)	0.0038 (3)	−0.0010 (4)
C16	0.0179 (5)	0.0155 (5)	0.0176 (5)	−0.0040 (4)	0.0031 (4)	0.0016 (4)
C17	0.0189 (5)	0.0136 (5)	0.0209 (5)	−0.0006 (4)	0.0017 (4)	−0.0034 (4)
C18	0.0179 (5)	0.0195 (5)	0.0164 (5)	0.0014 (4)	0.0042 (4)	−0.0050 (4)
C19	0.0142 (4)	0.0172 (5)	0.0130 (4)	−0.0003 (4)	0.0044 (4)	−0.0008 (4)
C20	0.0129 (4)	0.0145 (5)	0.0076 (4)	−0.0008 (3)	0.0027 (3)	0.0007 (3)
C21	0.0126 (4)	0.0142 (5)	0.0140 (4)	0.0004 (3)	0.0027 (3)	0.0027 (4)
C22	0.0123 (4)	0.0198 (5)	0.0171 (5)	0.0030 (4)	0.0043 (4)	0.0035 (4)
C23	0.0139 (4)	0.0213 (5)	0.0145 (5)	−0.0034 (4)	0.0052 (4)	0.0010 (4)
C24	0.0195 (5)	0.0143 (5)	0.0134 (4)	−0.0042 (4)	0.0076 (4)	−0.0010 (4)
C25	0.0153 (4)	0.0144 (5)	0.0119 (4)	−0.0008 (4)	0.0055 (3)	−0.0014 (4)
C26	0.0130 (4)	0.0124 (4)	0.0123 (4)	−0.0009 (3)	0.0034 (3)	−0.0021 (3)
C27	0.0173 (4)	0.0145 (5)	0.0132 (4)	0.0011 (4)	0.0050 (4)	−0.0013 (4)
C28	0.0231 (5)	0.0175 (5)	0.0133 (5)	0.0039 (4)	0.0029 (4)	0.0016 (4)
C29	0.0188 (5)	0.0229 (6)	0.0172 (5)	0.0069 (4)	−0.0003 (4)	−0.0014 (4)
C30	0.0132 (4)	0.0313 (6)	0.0200 (5)	0.0028 (4)	0.0042 (4)	−0.0013 (4)
C31	0.0148 (4)	0.0225 (5)	0.0161 (5)	0.0000 (4)	0.0045 (4)	0.0017 (4)
C32	0.0115 (4)	0.0134 (4)	0.0130 (4)	−0.0032 (3)	0.0050 (3)	−0.0009 (3)
C33	0.0145 (4)	0.0153 (5)	0.0171 (5)	−0.0019 (4)	0.0069 (4)	−0.0020 (4)
C34	0.0177 (5)	0.0206 (5)	0.0210 (5)	−0.0056 (4)	0.0113 (4)	−0.0079 (4)
C35	0.0224 (5)	0.0265 (6)	0.0130 (5)	−0.0098 (4)	0.0086 (4)	−0.0058 (4)
C36	0.0222 (5)	0.0204 (5)	0.0125 (4)	−0.0054 (4)	0.0039 (4)	0.0016 (4)
C37	0.0178 (5)	0.0140 (5)	0.0133 (4)	−0.0023 (4)	0.0041 (4)	−0.0004 (4)

### Geometric parameters (Å, º)

**Table:**

C1—N2	1.3427 (12)	B1—C14	1.6437 (15)
C1—N1	1.3445 (12)	B1—C26	1.6445 (15)
C1—N3	1.3453 (13)	B1—C20	1.6467 (14)
N1—C3	1.4600 (13)	B1—C32	1.6497 (14)
N1—C2	1.4632 (13)	C14—C15	1.4054 (13)
N2—C5	1.4628 (14)	C14—C19	1.4102 (13)
N2—C4	1.4660 (13)	C15—C16	1.3986 (15)
N3—C6	1.4639 (12)	C15—H15A	0.9500
N3—C7	1.4688 (12)	C16—C17	1.3902 (15)
C2—H2A	0.9800	C16—H16A	0.9500
C2—H2B	0.9800	C17—C18	1.3927 (15)
C2—H2C	0.9800	C17—H17A	0.9500
C3—H3A	0.9800	C18—C19	1.3928 (15)
C3—H3B	0.9800	C18—H18A	0.9500
C3—H3C	0.9800	C19—H19A	0.9500
C4—H4A	0.9800	C20—C21	1.4059 (13)
C4—H4B	0.9800	C20—C25	1.4065 (14)
C4—H4C	0.9800	C21—C22	1.3966 (13)
C5—H5A	0.9800	C21—H21A	0.9500
C5—H5B	0.9800	C22—C23	1.3882 (15)
C5—H5C	0.9800	C22—H22A	0.9500
C6—H6A	0.9800	C23—C24	1.3916 (14)
C6—H6B	0.9800	C23—H23A	0.9500
C6—H6C	0.9800	C24—C25	1.3933 (13)
C7—C8	1.5237 (14)	C24—H24A	0.9500
C7—H7A	0.9900	C25—H25A	0.9500
C7—H7B	0.9900	C26—C27	1.4000 (14)
C8—C9	1.5289 (14)	C26—C31	1.4090 (13)
C8—H8A	0.9900	C27—C28	1.3985 (14)
C8—H8B	0.9900	C27—H27A	0.9500
C9—N4	1.4600 (12)	C28—C29	1.3872 (15)
C9—H9A	0.9900	C28—H28A	0.9500
C9—H9B	0.9900	C29—C30	1.3906 (16)
N4—C11	1.3697 (13)	C29—H29A	0.9500
N4—C10	1.4597 (12)	C30—C31	1.3885 (15)
C10—H10A	0.9800	C30—H30A	0.9500
C10—H10B	0.9800	C31—H31A	0.9500
C10—H10C	0.9800	C32—C37	1.4055 (14)
C11—O1	1.2292 (13)	C32—C33	1.4069 (14)
C11—N5	1.3888 (13)	C33—C34	1.3985 (14)
N5—C12	1.4595 (15)	C33—H33A	0.9500
N5—C13	1.4624 (16)	C34—C35	1.3867 (17)
C12—H12A	0.9800	C34—H34A	0.9500
C12—H12B	0.9800	C35—C36	1.3913 (16)
C12—H12C	0.9800	C35—H35A	0.9500
C13—H13A	0.9800	C36—C37	1.3912 (14)
C13—H13B	0.9800	C36—H36A	0.9500
C13—H13C	0.9800	C37—H37A	0.9500
			
N2—C1—N1	119.65 (9)	H13A—C13—H13B	109.5
N2—C1—N3	119.57 (9)	N5—C13—H13C	109.5
N1—C1—N3	120.78 (9)	H13A—C13—H13C	109.5
C1—N1—C3	120.96 (9)	H13B—C13—H13C	109.5
C1—N1—C2	123.50 (8)	C14—B1—C26	105.98 (8)
C3—N1—C2	115.47 (8)	C14—B1—C20	111.87 (8)
C1—N2—C5	122.02 (8)	C26—B1—C20	112.94 (8)
C1—N2—C4	121.80 (9)	C14—B1—C32	112.29 (8)
C5—N2—C4	116.15 (8)	C26—B1—C32	110.67 (7)
C1—N3—C6	121.29 (8)	C20—B1—C32	103.25 (7)
C1—N3—C7	122.73 (8)	C15—C14—C19	115.07 (9)
C6—N3—C7	115.75 (8)	C15—C14—B1	123.97 (8)
N1—C2—H2A	109.5	C19—C14—B1	120.81 (8)
N1—C2—H2B	109.5	C16—C15—C14	122.61 (9)
H2A—C2—H2B	109.5	C16—C15—H15A	118.7
N1—C2—H2C	109.5	C14—C15—H15A	118.7
H2A—C2—H2C	109.5	C17—C16—C15	120.39 (10)
H2B—C2—H2C	109.5	C17—C16—H16A	119.8
N1—C3—H3A	109.5	C15—C16—H16A	119.8
N1—C3—H3B	109.5	C16—C17—C18	118.76 (10)
H3A—C3—H3B	109.5	C16—C17—H17A	120.6
N1—C3—H3C	109.5	C18—C17—H17A	120.6
H3A—C3—H3C	109.5	C17—C18—C19	120.04 (9)
H3B—C3—H3C	109.5	C17—C18—H18A	120.0
N2—C4—H4A	109.5	C19—C18—H18A	120.0
N2—C4—H4B	109.5	C18—C19—C14	123.07 (9)
H4A—C4—H4B	109.5	C18—C19—H19A	118.5
N2—C4—H4C	109.5	C14—C19—H19A	118.5
H4A—C4—H4C	109.5	C21—C20—C25	115.23 (8)
H4B—C4—H4C	109.5	C21—C20—B1	123.73 (9)
N2—C5—H5A	109.5	C25—C20—B1	120.68 (8)
N2—C5—H5B	109.5	C22—C21—C20	122.71 (9)
H5A—C5—H5B	109.5	C22—C21—H21A	118.6
N2—C5—H5C	109.5	C20—C21—H21A	118.6
H5A—C5—H5C	109.5	C23—C22—C21	120.29 (9)
H5B—C5—H5C	109.5	C23—C22—H22A	119.9
N3—C6—H6A	109.5	C21—C22—H22A	119.9
N3—C6—H6B	109.5	C22—C23—C24	118.72 (9)
H6A—C6—H6B	109.5	C22—C23—H23A	120.6
N3—C6—H6C	109.5	C24—C23—H23A	120.6
H6A—C6—H6C	109.5	C23—C24—C25	120.30 (10)
H6B—C6—H6C	109.5	C23—C24—H24A	119.9
N3—C7—C8	112.80 (8)	C25—C24—H24A	119.9
N3—C7—H7A	109.0	C24—C25—C20	122.75 (9)
C8—C7—H7A	109.0	C24—C25—H25A	118.6
N3—C7—H7B	109.0	C20—C25—H25A	118.6
C8—C7—H7B	109.0	C27—C26—C31	115.08 (9)
H7A—C7—H7B	107.8	C27—C26—B1	125.48 (8)
C7—C8—C9	112.45 (8)	C31—C26—B1	119.37 (8)
C7—C8—H8A	109.1	C28—C27—C26	122.74 (9)
C9—C8—H8A	109.1	C28—C27—H27A	118.6
C7—C8—H8B	109.1	C26—C27—H27A	118.6
C9—C8—H8B	109.1	C29—C28—C27	120.17 (10)
H8A—C8—H8B	107.8	C29—C28—H28A	119.9
N4—C9—C8	111.85 (8)	C27—C28—H28A	119.9
N4—C9—H9A	109.2	C28—C29—C30	118.91 (10)
C8—C9—H9A	109.2	C28—C29—H29A	120.5
N4—C9—H9B	109.2	C30—C29—H29A	120.5
C8—C9—H9B	109.2	C31—C30—C29	120.00 (10)
H9A—C9—H9B	107.9	C31—C30—H30A	120.0
C11—N4—C10	123.81 (8)	C29—C30—H30A	120.0
C11—N4—C9	119.24 (8)	C30—C31—C26	123.08 (10)
C10—N4—C9	115.85 (8)	C30—C31—H31A	118.5
N4—C10—H10A	109.5	C26—C31—H31A	118.5
N4—C10—H10B	109.5	C37—C32—C33	115.18 (9)
H10A—C10—H10B	109.5	C37—C32—B1	121.13 (8)
N4—C10—H10C	109.5	C33—C32—B1	123.37 (9)
H10A—C10—H10C	109.5	C34—C33—C32	122.47 (10)
H10B—C10—H10C	109.5	C34—C33—H33A	118.8
O1—C11—N4	122.44 (9)	C32—C33—H33A	118.8
O1—C11—N5	121.90 (10)	C35—C34—C33	120.34 (10)
N4—C11—N5	115.64 (9)	C35—C34—H34A	119.8
C11—N5—C12	115.71 (10)	C33—C34—H34A	119.8
C11—N5—C13	120.29 (9)	C34—C35—C36	118.91 (10)
C12—N5—C13	113.87 (10)	C34—C35—H35A	120.5
N5—C12—H12A	109.5	C36—C35—H35A	120.5
N5—C12—H12B	109.5	C37—C36—C35	120.00 (10)
H12A—C12—H12B	109.5	C37—C36—H36A	120.0
N5—C12—H12C	109.5	C35—C36—H36A	120.0
H12A—C12—H12C	109.5	C36—C37—C32	123.09 (10)
H12B—C12—H12C	109.5	C36—C37—H37A	118.5
N5—C13—H13A	109.5	C32—C37—H37A	118.5
N5—C13—H13B	109.5		
			
N2—C1—N1—C3	33.82 (13)	C26—B1—C20—C21	−143.51 (9)
N3—C1—N1—C3	−146.25 (9)	C32—B1—C20—C21	96.93 (10)
N2—C1—N1—C2	−143.10 (9)	C14—B1—C20—C25	163.19 (8)
N3—C1—N1—C2	36.83 (13)	C26—B1—C20—C25	43.71 (12)
N1—C1—N2—C5	−143.09 (10)	C32—B1—C20—C25	−75.85 (10)
N3—C1—N2—C5	36.98 (13)	C25—C20—C21—C22	−0.26 (14)
N1—C1—N2—C4	35.05 (13)	B1—C20—C21—C22	−173.40 (9)
N3—C1—N2—C4	−144.88 (9)	C20—C21—C22—C23	0.45 (15)
N2—C1—N3—C6	32.16 (14)	C21—C22—C23—C24	−0.24 (15)
N1—C1—N3—C6	−147.77 (9)	C22—C23—C24—C25	−0.13 (15)
N2—C1—N3—C7	−153.50 (9)	C23—C24—C25—C20	0.31 (15)
N1—C1—N3—C7	26.57 (14)	C21—C20—C25—C24	−0.12 (14)
C1—N3—C7—C8	124.04 (10)	B1—C20—C25—C24	173.24 (9)
C6—N3—C7—C8	−61.33 (11)	C14—B1—C26—C27	−103.76 (10)
N3—C7—C8—C9	−63.95 (10)	C20—B1—C26—C27	19.06 (13)
C7—C8—C9—N4	−63.61 (10)	C32—B1—C26—C27	134.25 (10)
C8—C9—N4—C11	115.84 (10)	C14—B1—C26—C31	73.21 (11)
C8—C9—N4—C10	−75.68 (11)	C20—B1—C26—C31	−163.97 (9)
C10—N4—C11—O1	−156.85 (10)	C32—B1—C26—C31	−48.77 (12)
C9—N4—C11—O1	10.66 (15)	C31—C26—C27—C28	1.18 (15)
C10—N4—C11—N5	21.14 (14)	B1—C26—C27—C28	178.27 (10)
C9—N4—C11—N5	−171.34 (9)	C26—C27—C28—C29	0.07 (16)
O1—C11—N5—C12	10.68 (15)	C27—C28—C29—C30	−1.18 (17)
N4—C11—N5—C12	−167.32 (9)	C28—C29—C30—C31	0.97 (17)
O1—C11—N5—C13	−132.58 (11)	C29—C30—C31—C26	0.38 (18)
N4—C11—N5—C13	49.42 (13)	C27—C26—C31—C30	−1.41 (16)
C26—B1—C14—C15	−94.51 (10)	B1—C26—C31—C30	−178.69 (10)
C20—B1—C14—C15	142.00 (9)	C14—B1—C32—C37	41.83 (12)
C32—B1—C14—C15	26.43 (13)	C26—B1—C32—C37	160.03 (9)
C26—B1—C14—C19	80.96 (10)	C20—B1—C32—C37	−78.85 (10)
C20—B1—C14—C19	−42.53 (12)	C14—B1—C32—C33	−145.01 (9)
C32—B1—C14—C19	−158.10 (8)	C26—B1—C32—C33	−26.81 (12)
C19—C14—C15—C16	2.36 (14)	C20—B1—C32—C33	94.31 (10)
B1—C14—C15—C16	178.07 (9)	C37—C32—C33—C34	0.24 (14)
C14—C15—C16—C17	−0.62 (16)	B1—C32—C33—C34	−173.29 (9)
C15—C16—C17—C18	−1.42 (16)	C32—C33—C34—C35	−0.30 (15)
C16—C17—C18—C19	1.58 (16)	C33—C34—C35—C36	−0.23 (15)
C17—C18—C19—C14	0.30 (16)	C34—C35—C36—C37	0.79 (15)
C15—C14—C19—C18	−2.21 (14)	C35—C36—C37—C32	−0.87 (16)
B1—C14—C19—C18	−178.07 (9)	C33—C32—C37—C36	0.34 (14)
C14—B1—C20—C21	−24.04 (13)	B1—C32—C37—C36	174.03 (9)

### Hydrogen-bond geometry (Å, º)

**Table:**

*D*—H···*A*	*D*—H	H···*A*	*D*···*A*	*D*—H···*A*
C13—H13*C*···O1^i^	0.98	2.67	3.395 (2)	131

Symmetry code: (i) −*x*+1/2, *y*+1/2, −*z*+1/2.
